# The development of quality indicators in mental healthcare: a discrete choice experiment

**DOI:** 10.1186/1471-244X-12-103

**Published:** 2012-08-07

**Authors:** Ron Schellings, Brigitte AB Essers, Alfons G Kessels, Florian Brunner, Tijmen van de Ven, Paul BM Robben

**Affiliations:** 1 Health Care Inspectorate; Ministry of Health, Welfare, and Sports, Den Bosch, The Netherlands; 2 Department of Clinical Epidemiology and Medical Technology Assessment, Maastricht University Medical Centre, P.O. Box 5800, 6202 AZ Maastricht, the Netherlands; 3 Horten Centre, Zürich University, Zürich, Switzerland; 4 Department of Physical Medicine and Rehabilitation, Balgrist University Clinic, Zürich, Switzerland; 5 Institute of Healthcare Policy & Management, Erasmus University, Rotterdam, the Netherlands

**Keywords:** Discrete choice experiment, Healthcare regulatory agencies, Quality indicators, Mental healthcare, Quality of care

## Abstract

**Background:**

Health care regulatory agencies perform audits or inspections to judge the quality and safety of health care. This judgment is based on the assessment of a large set of health care indicators as accepted by the profession. However, there is a lack of knowledge about the influence of these indicators and whether a smaller number would be sufficient for a quality assessment or audit procedure.

**Methods:**

A discrete choice experiment (DCE) was performed for the assessment of quality of care regarding the management of patients with schizophrenia and drug dependency in psychiatric institutes. Based on multidisciplinary guidelines for the treatment of schizophrenia and a visit of (co)inspectors of the Dutch Healthcare Inspectorate at all 33 integrated mental hospitals a set of 51 indicators were assessed in a subsequent interview. With the analysis of the results, 6 attributes were selected for the DCE as quality indicators.

**Results:**

Seventy-six percent of all health services (co)inspectors (n = 33) involved in the inspection of mental health services, participated in the experiment. Respondents considered an operational elaborate treatment plan the most important indicator for the assessment of quality of care in a psychiatric institute, followed by a general care program, treatment outcome measurement, and involvement in treatment of patients and relatives. Pharmacotherapy and governance responsibility were valued as less important indicators.

**Conclusions:**

The results of this DCE show that there is a prioritisation in the six selected quality indicators. This might help health services (co) inspectors to enhance the efficiency and transparency of the quality of care assessment for patients with schizophrenia and/or drug dependency in psychiatric institutes.

## Background

Health care regulatory agencies perform audits or inspections to judge the quality and safety of healthcare. This judgment is usually based on the assessment of a large set of healthcare indicators. However, there is a lack of knowledge about the relative importance of these indicators and whether a smaller number would be sufficient for an audit procedure [1]. To rank indicators with respect to their importance and enhance the transparency of the quality of care assessment, several approaches can be applied such as a survey, a Delphi method or different variants of conjoint analyses. However, most of these methods might lead to a result where different indicators are considered equally highly important and consequently no information is provided about the order of their relative importance. A discrete choice experiment (DCE) is a technique within the field of health care that may have an advantage compared to the traditional survey or Delphi method [2]. A DCE is an attribute based survey method for measuring benefits or utility [3]. It involves presenting respondents with a number of choice sets which consist of two or more hypothetical alternatives that differ in levels of various attributes. Every time a choice-set is offered, respondents are asked to choose their preferred alternative. As such, a discrete choice experiment forces respondents to make a tradeoff between the levels of the different attributes when choosing an alternative. A crucial step in this experiment is the choice of the attributes which will determine to a great extent the applicability of the results.

In this study, we applied the technique of the discrete choice experiment to investigate which indicators have the greatest impact on the decisions of health services inspectors concerning the assessment of quality of care for patients with schizophrenia and drug dependency.

## Methods

In the design and the development of this DCE, we were guided by the studies of Ryan & Farrar and Lancsar & Louviere [4,5].

### Establishing attributes

The selection of the attributes is based on 51 indicators that were selected by an independent research institute from a multidisciplinary guideline for the treatment of schizophrenia, a guideline for the treatment of patients with double-diagnosis, general guidelines to judge the quality of patient records, and health care legislations [6-9]. In 2008 pairs of inspectors and co-inspectors visited all 33 integrated psychiatric institutes in the Netherlands. In total 14 inspectors and four co-inspectors assessed, for each hospital, on a four level scale the extent to which 51 indicators were operational: no policy with respect to the specific indicator is apparent (level 0), there is a policy with respect to the specific indicator and all employees are well informed about the policy (level 1), the policy is fully applied (level 2) and the effects of the policy are periodically evaluated (level 3) [10].

We grouped these 51 indicators into ten clusters based on content association and within each cluster the outcomes of the indicator assessment were added up. Furthermore, we asked each pair of inspectors and co-inspectors to judge whether sufficient care in the attended hospitals was present or not. A logistic regression analysis with this outcome as the dependent variable and the sum score of each cluster as independent variable resulted in ten weights of the indicator clusters. Based on this analysis, we selected the seven clusters with the best predictive power for the ‘sufficient care‘ judgment as outcome. These seven clusters were presented to the visiting inspectors and co-inspectors and they were asked to rank the three most important and the three least important clusters based on their professional experience. In addition, they were enabled to add other issues that might be important.

We selected the six clusters with the highest score and included them as attributes for the DCE. Table 1 shows the attributes and the definitions.

**Table 1:** Definitions of attributes

**Attribute**	**Definition**
Treatment plan	An elaborate plan for the individual patient including information about psychiatric diagnosis, informed consent, goals of treatment, therapeutic interventions.
Care program	A general program in which a comprehensive view on the treatment of patients is presented.
Treatment outcome measurement	Treatment outcome is periodically measured by means of a standardised method and results are used for the revision of the individual treatment plan and for the purpose of the aggregation of treatment results.
Involvement in treatment of patients and relatives	The treatment plan is determined in consultation with the patient and his or her relatives.
Pharmacotherapy	Pharmacotherapy is based on medication guidelines.
Governance responsibility	The governing body is informed about the quality of care and will adjust the policy.

### Establishing levels

For each of these six clusters we defined two levels: operational and not operational. Operational was defined as fulfilling all three afore mentioned conditions i.e. all employees are well informed about the policy on a care attribute, the policy is fully applied and the effects of the policy are periodically evaluated, not operational was defined as no policy apparent.

We chose for these two levels as we considered this as unambiguous for the respondents. In addition, we wanted to keep the description of the six attributes and the two levels cognitively acceptable and manageable.

### Experimental design

Since no prior estimates of the utility related to the attributes (e.g. based on literature or previous research) were available, a scenario was composed of three attributes with a desirable level (operational) and three attributes with a not desirable level (not operational) although this could lead to a less optimal design. Subsequently, each scenario was paired with a scenario consisting of complementary levels of all attributes (i.e. a foldover design). With six attributes, each having two levels, it is possible to construct 10 choice-sets (with different pairs of scenarios). Table 2 shows an example of a choice-set. As contextual information, it was stated that the delivery of care and the organisation of the institute were the same in all remaining aspects. The order of the attributes within the scenarios was randomly assigned to each respondent, in such way that no respondent was offered the same order. The choice-sets were presented in a web-based questionnaire.

**Table 2:** Example of a choice-set

**Attributes/**	**Psychiatric institute A**	**Psychiatric institute B**
Treatment plan	Operational	Not operational
Involvement in treatment of patients and relatives	Not operational	Operational
Care program	Operational	Not operational
Treatment outcome measurement	Operational	Not operational
Pharmacotherapy	Not operational	Operational
Governance responsibility	Not operational	Operational
Which hospital do you choose?	A	B

### Pilot

We tested the choice-sets with four employees of the Dutch Healthcare Inspectorate who were supervising the process of inspection. This pilot showed that they understood and accepted the DCE, that completing the web-based questionnaire took about 15 minutes and was not experienced as burdensome. Furthermore, the psychiatric institutes were according to these employees sufficiently characterized by the scenarios.

### Data collection

Thirty-three employees of the Healthcare Inspectorate, who were involved in the inspection of mental health care services, were asked to participate in the discrete choice experiment. It concerned 18 inspectors with experience in patient care, 8 co-inspectors with a background in healthcare, 4 legal advisors, and 3 advising psychiatrists. They received the web-based questionnaire with general information and instructions. The questionnaire consisted of 10 choice-sets with unlabeled descriptions of two mental health care hospitals which differed in the levels of the attributes. Each time a choice-set was offered, the participants i.e. employees of the Healthcare Inspectorate, had to choose which institute, A or B, provided the best quality of care. We used an unlabelled generic design, which means that the labels attached to each option convey no information beyond that provided by the attributes [11].

### Statistical analysis

The analysis was performed using STATA version 11 (StataCorp, College Station, Texas). The weights of the attributes were determined with a logistic regression model for panel data with the choice of the respondents as dependent variable and the attributes as independent variables. We used effect coding with the levels of the attributes set to −1 or 1 with constant set to zero. As the sum of the attributes will be zero for all scenarios, one attribute will be left out because of collinearity and will be the reference. The exponent of the coefficient of a specific attribute in the model can be interpreted as the relative influence on the decision as compared to the influence of the reference attribute that was left out.

## Results

### Response

25 employees (76% of the total population) participated in the DCE consisting of 13 inspectors, 7 co-inspectors, 2 legal advisors, and 3 advising psychiatrists. The duration of work experience within the Inspectorate ranged from one to twenty eight years with a median of six years (25% percentile =2; 75% percentile = 12). The 8 employees, who did not respond, did not significantly differ with regard to the duration of work experience compared to the respondents.

### Weights of attributes

Table 3 gives an overview of the relative weights of the attributes. The results can be interpreted as a ranking of the importance of the attributes. This means that respondents considered an elaborate treatment plan significantly, 1.8 to 3.7 times, more important for the assessment of the best quality of care regarding patients with schizophrenia than the other attributes. The second significantly important attribute was a general care program, followed by treatment outcome measurement and involvement in treatment of patients and relatives. Pharmacotherapy and governance responsibility seemed to be less important indicators for assessing the quality of care in a psychiatric institute.

**Table 3:** Odds Ratio’s as measure of the relative importance between the attributes

**Attributes**	**Governance responsibility**	**Pharmaco-therapy**	**Involvement in treatment**	**Outcome measurement**	**Care program**
Plan of treatment	3.7^§^	3.2^§^	2.6^§^	2.1^§^	1.8^§^
Care program	2.0^§^	1.7^§^	1.4	1.1	
Outcome measurement	1.8^§^	1.5^¶^	1.2		
Involvement in treatment	1.5^¶^	1.2			
Pharmaco-therapy	1.2				

§: p < 0.01, ¶: p < 0.05.

## Discussion

The primary objective of this study was to examine which indicators have the largest impact on the inspectors’ decisions regarding the assessment of quality of care for patients with schizophrenia and drug dependency in psychiatric institutes. For that purpose, we applied the technique of the discrete choice experiment (DCE) among health services inspectors. From the perspective of the Dutch Healthcare Inspectorate, respondents considered an operational elaborate plan as the most important, followed by a care program, measurement of treatment outcomes and the involvement of patients and relatives in the treatment. Pharmacotherapy and governance responsibility were valued as less important for the assessment of quality of care. This information might be useful to enhance the transparency and efficiency of the quality assessment process for both regulatory agencies and hospitals. In addition, it shows that it is possible to use a limited number instead of a large set of indicators in an assessment or audit process.

A strength of this study is that the selection of respondents consisted of the entire population of inspectors who are involved in the regulation of institutes providing care to patients with schizophrenia. This means that in total 33 persons were invited to participate in the discrete choice experiment. Although 8 persons did not respond, their characteristics did not differ from those respondents who participated in the DCE.The selection of attributes, levels, and the amount of scenarios is a process of weighing a sound methodology against the cognitive burden and complexity for the respondent [12]. In our study, the attributes were carefully selected from general accepted guidelines and a survey among employees of the Healthcare Inspectorate. In this way the amount of attributes was limited in a well-considered way. In addition, the DCE was tested with a pilot study among employees who were supervising the process of inspection.

In the development of quality indicators more parties should be involved such as the patients themselves, their family and the caregivers. Besides, the development of quality indicators for healthcare needs an international approach [13,14].

New developed indicators are intended to be applied by the inspectorate. Moreover the inspectorate decides whether institutes fail in their care and whether they should draw up a plan for improvement. In this way, their view can be considered as a reference for the assessment of quality and will have a direct influence on the management of the institutes.

In recent years, a number of studies used the technique of the DCE for different applications. For example, to examine patient preferences for a service or treatment, to determine the strength of hospital consultants’ preferences for various aspects of their work or to identify which criteria as presented in Health Technology assessment (HTA) studies are important for decision makers in health care priority setting [15-20]. To the best of our knowledge, this is the first time that a DCE is conducted among employees of a regulatory agency with the aim to examine which indicators are considered the most important for the assessment of quality of care. The results of this DCE might contribute to the development of uniform and consistent guidelines for inspections performed by regulatory agencies. In addition, this enables them to provide feedback to health services and institutions under regulation and in this way improve their performance.

Our DCE was restricted to the regulation of mental health care, in particular the quality of care for patients with schizophrenia and drug dependency. However, this method is also applicable to (all the) other fields of health care from a regulation point of view. Besides, we believe the DCE method can be generally applied in governance and management of health care.

## Conclusions

Results from DCEs, performed on organisational and regulatory levels, can contribute to the development of quality indicators with joint interests for both management of psychiatric institutes and regulatory agencies. Finally, our study shows that a discrete choice experiment is an informative technique that can help health care regulating agencies to identify the most important indicators and enhance the transparency and efficiency of the assessment process with respect to the quality of care in health care institutions.

## Competing interests

The authors declare that they have no competing interests.

## Authors’ contributions

RS participated in the design, the coordination of the study, interpretation of the results and drafting of the manuscript. BABE participated in the design, the interpretation of the results and the drafting of the manuscript. FK participated in the design, performed the statistical analysis, interpretation of results and drafting of the manuscript. FB, TvdV and PR helped with the coordination of the study and drafting of the manuscript. All authors read and approved the final manuscript.

## Author details

^1^Health Care Inspectorate; Ministry of Health, Welfare, and Sports, Den Bosch, The Netherlands. ^2^Department of Clinical Epidemiology and Medical Technology Assessment, Maastricht University Medical Centre, P.O. Box 5800, 6202 AZ Maastricht, the Netherlands. ^3^Horten Centre, Zürich University,Zürich, Switzerland. ^4^Department of Physical Medicine and Rehabilitation, Balgrist University Clinic, Zürich, Switzerland. ^5^Institute of Healthcare Policy & Management, Erasmus University, Rotterdam, the Netherlands.

## Pre-publication history

The pre-publication history for this paper can be accessed here:

http://www.biomedcentral.com/1471-244X/12/103/prepub
